# Social appearance anxiety and aggressive behavior in children with dentofacial deformities: a mediation and profile analysis of family support and self-stigma

**DOI:** 10.3389/fpubh.2026.1914699

**Published:** 2026-09-14

**Authors:** Wenjun Wang, Jingjing Zhang, Bin Xia, Junxia Zhu

**Affiliations:** Department of Pediatric Dentistry, Peking University School and Hospital of Stomatology, National Center of Stomatology, National Clinical Research Center for Oral Diseases, National Engineering Research Center for Oral Biomaterials and Digital Medical Devices, Beijing, China

**Keywords:** aggressive behavior, children, dentofacial deformities, family support, latent profile analysis, self-stigma, social appearance anxiety

## Abstract

**Objective:**

To examine the relationship between social appearance anxiety and aggressive behavior in children with dentofacial deformities, to investigate the mediating role of self-stigma and the moderating role of family support within this relationship, and to identify heterogeneous subgroups of social appearance anxiety and self-stigma through a person-centered approach.

**Methods:**

A cross-sectional survey design was employed. A total of 482 children diagnosed with dentofacial deformities were recruited from Peking University School and Hospital of Stomatology between September 2025 and March 2026. Participants completed validated Chinese versions of the Social Appearance Anxiety Scale, the Self-Stigma subscale, the Family Support subscale, and the Aggression Scale.

**Results:**

Social appearance anxiety was significantly and positively associated with aggressive behavior (*B* = 0.129, *p* = 0.007). Self-stigma showed a significant indirect statistical association between social appearance anxiety and aggressive behavior [indirect effect = 0.352, 95% CI (0.233, 0.482)]. Family support was a significant statistical moderator of both the direct and indirect pathways: the cross-sectional direct effect of social appearance anxiety on aggressive behavior was attenuated and became non-significant at high levels of family support, and the index of moderated mediation was significant [−0.020, 95% bootstrap CI (−0.038, −0.005)], indicating that higher family support weakened the cross-sectional indirect effect transmitted through self-stigma. LPA identified three distinct latent profiles: a low social appearance anxiety–self-stigma group (51.8%), a moderate social appearance anxiety–self-stigma group (36.5%), and a high social appearance anxiety–self-stigma group (11.7%). The three profiles differed significantly in both aggressive behavior (*F* = 113.374, *p* < 0.001) and family support (*F* = 3.480, *p* = 0.032), with the high-profile group exhibiting the most elevated levels of aggressive behavior.

**Conclusion:**

These findings highlight the imperative of integrating psychological assessment and family-centered intervention into the clinical care of children with dentofacial deformities.

## 1 Introduction

Dentofacial deformity refers to a disharmony in the morphology, position, or function of the teeth, jaws, and facial structures resulting from aberrant jaw development (1, 2). Prior research has linked dentofacial deformities to familial inheritance, embryonic developmental anomalies, metabolic and endocrine dysfunctions, and deleterious oral habits (3, 4), while scant attention has been paid to the impact of dentofacial deformities on facial aesthetics and psychosocial wellbeing. This gap is critically important, because by disrupting the harmony of the jaws, dentition, and soft tissues, dentofacial deformities produce facial disproportionality and asymmetry (5, 6), thereby intensifying the individual's social appearance anxiety. Social appearance anxiety is a negative emotional state characterized by persistent worry, tension, and low self-esteem. It commonly manifests as self-derogation, social avoidance, and body image disturbance (7, 8), and is particularly pronounced among pediatric patients aged 0–18 years. On the one hand, children whose appearance elicits peer ridicule may internalize their dentofacial deformity as a self-deficiency label, engendering self-stigma (9); on the other hand, problems such as slurred speech, social withdrawal, and peer exclusion mean that affected children frequently experience marginalization stress during social interactions (10). Recent research has identified a correlation between social appearance anxiety and self-esteem (11). However, that study did not extend to examining the relationship between social appearance anxiety and aggressive behavior, nor has this relationship been investigated in children with dentofacial deformities. Therefore, exploring the relationship between social appearance anxiety and aggressive behavior in children with dentofacial deformities carries substantial practical significance.

Aggressive behavior refers to an individual's or a group's intentional infliction of physical, verbal, or psychological harm upon others (12, 13). Aggressive behavior in children has emerged as a prominent research focus within the field of child mental health in recent years (14, 15). A review of the extant literature reveals that studies on aggressive behavior have concentrated chiefly on domains such as family structure, parenting style, educational environment, and cognitive-emotional processes (16, 17). For example, Zhang, Cao (18) demonstrated that exposure to violent video games increases children's aggressive cognition and aggressive behavior. Le, Huynh (19) reported a significant indirect association between children's personality traits and aggressive behavior. These two studies analyzed the antecedents of aggressive behavior from the perspectives of personality traits and external stimuli, respectively, yet neither attended to children's facial appearance characteristics. Put differently, prior research has not extended to examining the relationship between children's social appearance anxiety and aggressive behavior, particularly among children with dentofacial deformities. This oversight is regrettable, insofar as children who are highly concerned about their appearance are likely to encounter peer rejection or ridicule, which may engender frustration and anger that manifest as aggressive behavior. This association underscores the imperative to investigate the psychological mechanisms linking social appearance anxiety to behavioral outcomes in children with dentofacial deformities. Accordingly, the present study seeks to address the following two questions:

RQ1: What is the effect of social appearance anxiety on aggressive behavior in children with dentofacial deformities?

RQ2: What are the underlying mechanisms and boundary conditions of this relationship?

### 1.1 Variable-centered perspective

Self-stigma is a process of self-devaluation in which an individual internalizes society's negative stereotypes regarding a particular identity (20). Extant research on self-stigma has centered predominantly on individuals with dental and facial deformities (21). Specifically, the cleft lip, facial asymmetry, dental protrusion, and other visible irregularities resulting from dentofacial deformities may serve as salient sources of self-stigma (22). According to the psychosocial pathway model (23), self-stigma operates through an internalization process whereby individuals develop excessive preoccupation with and negative appraisal of their appearance defects, thereby engendering social appearance anxiety. This anxiety state may, in turn, influence behavioral expression through multiple pathways, including an increase in aggressive behavior (24). Concurrently, self-stigma may diminish individuals‘ psychological resources for coping with appearance defects by undermining their self-efficacy and self-esteem (25). Such eroded self-efficacy may precipitate aggressive behavior as a result of the frustration and anger individuals experience when confronting external provocation. Thus, by shaping patients' negative self-evaluations, emotional states, and social-cognitive capacities, self-stigma plays a pivotal mediating role in the relationship between social appearance anxiety and aggressive behavior in children with dentofacial deformities.

Family support refers to the provision of emotional, informational, evaluative, and instrumental resources and companionship by family members (26, 27). Hartup (28) first proposed that family support constitutes the core factor in children's psychological health development, capable of shaping children's self-cognition and social adaptive capacity through emotional companionship and social support. Particularly among children facing appearance defects such as dentofacial deformities, family support provides not only emotional solace but also enhanced self-cognition and social cognition, thereby helping children better cope with external challenges (29, 30). Research has demonstrated that family support can significantly alleviate the psychological distress arising from children's appearance defects (31). Drawing on self-determination theory (32), family support fulfills children's basic psychological needs, thereby promoting their mental health and behavioral adaptation. Among children with dentofacial deformities, family support helps them overcome the psychological distress brought about by appearance defects by satisfying their needs for relatedness and competence. Family support can assist children in reconstructing a positive self-image and attenuating self-stigmatizing cognitions, thereby reducing aggressive behavior triggered by social appearance anxiety. Consequently, family members can help children with dentofacial deformities reconstruct their self-cognition, alleviate social appearance anxiety, and diminish the occurrence of aggressive behavior.

Drawing on the foregoing analysis, the present study advances the following hypotheses:

**H1:** Social appearance anxiety is significantly and positively associated with aggressive behavior in children with dentofacial deformities.

**H2:** Self-stigma serves as a statistical mediator in the relationship between social appearance anxiety and aggressive behavior in children with dentofacial deformities.

**H3:** Family support cross-sectional moderates the relationship between social appearance anxiety and self-stigma.

### 1.2 Person-centered perspective

Drawing on the cognitive-behavioral framework (33), Cash, Melnyk (34) defined social appearance anxiety as the distress arising from excessive preoccupation with specific physical appearance features. However, a person-centered perspective reveals deeper heterogeneity. Notably, cultural factors may also produce differentiation through individual internalization mechanisms. For instance, East Asian populations, influenced by face culture, are more prone to channel their anxiety toward skin whiteness (35). Consequently, when assessed using the same scale, working women may score highly on items concerning fear of being scrutinized, while individuals with body dysmorphic disorder may exhibit pathologically stable traits on items concerning imagined defects. Such individual differences call for researchers to move beyond group-mean analyses and employ techniques such as latent profile analysis (LPA) to capture heterogeneous subgroups. Self-stigma is defined as a form of internalized social evaluation characterized by self-derogation, social avoidance, and emotional distress (36, 37). When individuals perceive others' negative evaluations of their appearance defects, they may develop self-derogatory cognitions and negative emotional states; conversely, when individuals perceive positive evaluation and support from others, the formation of self-stigma may be attenuated.

Nevertheless, the relationship between social appearance anxiety and self-stigma exhibits a perplexing bidirectional paradox. Prior research has demonstrated that social appearance anxiety can positively predict self-stigma (38, 39). Yet research on cosmetic surgery populations has revealed a reverse pathway, wherein patients' anxiety declines postoperatively while their self-stigma paradoxically intensifies (40). The inconsistency in findings regarding the relationship between social appearance anxiety and self-stigma may stem from the limitations of prior research perspectives. The majority of existing studies have adopted a variable-centered perspective, which frequently overlooks individual differences. The person-centered approach aims to reveal within-population heterogeneity by identifying subgroups that share similar relationships, levels, or patterns within a given variable system. The LPA is a commonly employed person-centered analytical method that partitions participants into mutually exclusive groups based on their response patterns to measurement items (41), such that individuals within the same group share similar characteristics and response profiles.

Therefore, the present study first examines the relationship between social appearance anxiety and aggressive behavior in children with dentofacial deformities from a variable-centered perspective, and further analyzes the underlying mechanism linking the two constructs.

The research framework of the present study is depicted in Figure 1.

**Figure 1:**
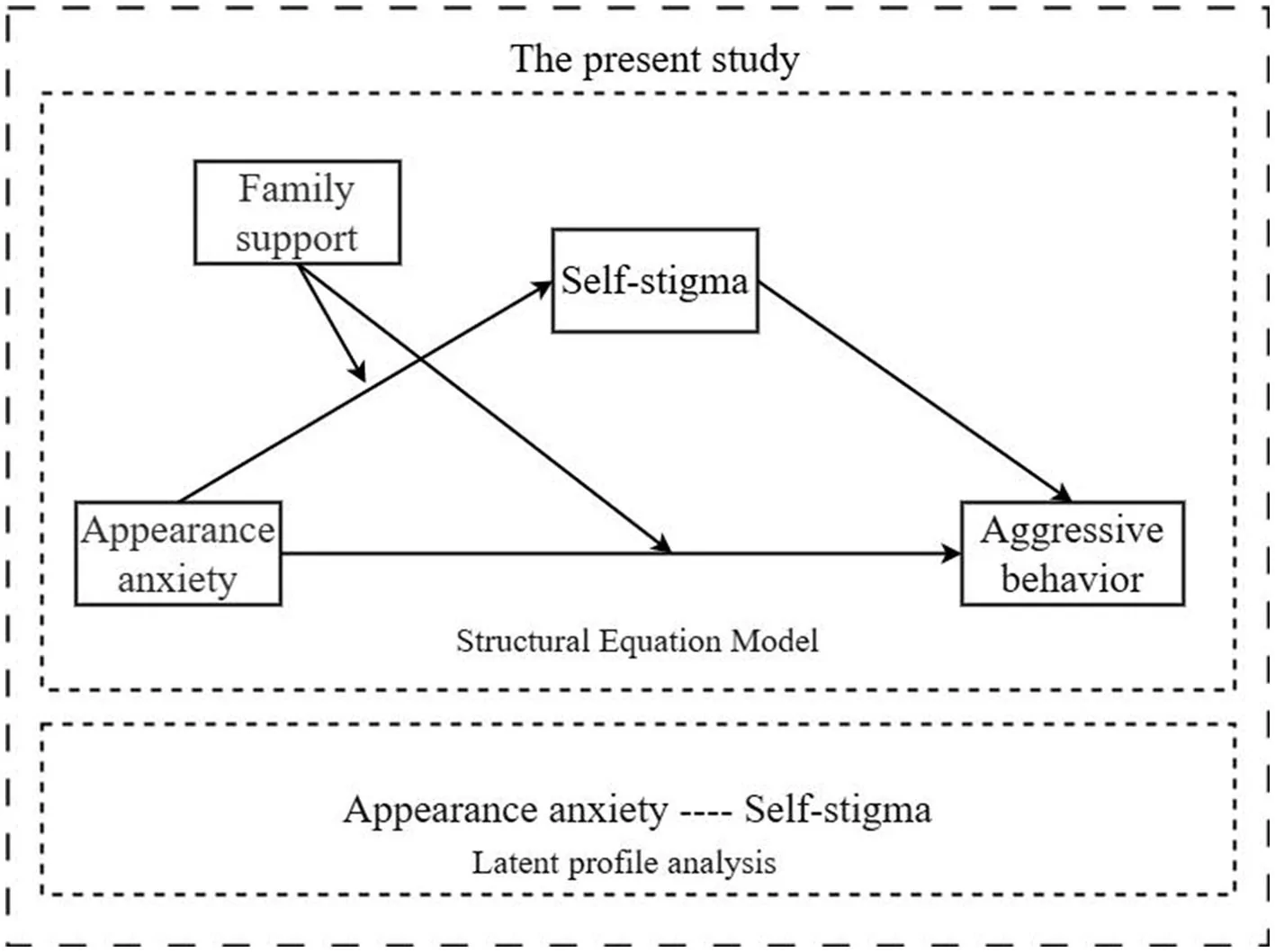
Study framework.

## 2 Method

This study employed a cross-sectional research design and adopted a dual perspective integrating person-centered and variable-centered approaches to examine the relationship between social appearance anxiety and aggressive behavior in children with dentofacial deformities, and to further analyze the heterogeneity of social appearance anxiety and self-stigma. Informed consent was obtained from all participants and their legal guardians, and the study was reviewed and approved by the Institutional Review Board of Peking University and Hospital of Stomatology (No.: PKUSSIRB-2025114169). Because the study is cross-sectional, the mediation and moderation analyses test statistical associations rather than temporal or causal relationships; bidirectional or reverse pathways are possible and should be tested in future longitudinal designs.

### 2.1 Participants

#### 2.1.1 Recruitment procedure

Data were collected between September 20, 2025, and March 12, 2026, at Peking University and Hospital of Stomatology in Beijing, China. The survey questionnaire was administered via a professional data collection platform (Credamo) and was available in both electronic and paper-based formats, with the electronic version presented as a QR code. The researchers first contacted the chief physicians of the Department of Pediatric Dentistry and the Department of Oral and Maxillofacial Surgery at Peking University and Hospital of Stomatology and explained the study objectives, potential risks, and other relevant details in full. Upon obtaining their approval, paper-based questionnaires were distributed to patients who had difficulty using mobile devices, while electronic questionnaires were provided to patients capable of using such devices. Prior to their formal participation in the survey, all patients were informed of the study's purpose, procedures, potential benefits, and the voluntary nature of their participation. Patients proceeded to complete the survey only after providing informed consent. For participants who were unable to recognize all Chinese characters used in the questionnaire, the researchers provided objective explanations without interfering with their responses. Additionally, professional psychological counselors were made available on site to provide psychological support as needed.

#### 2.1.2 Minimum sample size estimation

G^*^Power software (version 3.1) was used to determine the minimum required sample size for the present study. An F-test was selected, with the statistical test specified as “Linear multiple regression: Fixed model, *R*^2^ deviation from zero,” an effect size *f* ^2^ = 0.15, a significance level of α = 0.05, and a statistical power of 1 – β = 0.95. The results indicated that a minimum sample size of 129 participants was required.

**Exclusion criteria** for this study were as follows: (1) patients with severe mental disorders or cognitive impairment; (2) facial deformities of non-dentofacial origin; (3) presence of language or communication barriers; (4) duplicate participation or research fatigue; (5) patients younger than 6 years or older than 18 years; (6) absence of a confirmed diagnosis of dentofacial deformity by an orthodontic specialist; (7) survey completion time of less than 6 min or a response pattern exhibiting excessively uniform consistency.

Duplicate participation or research fatigue“ was defined and assessed as follows. Duplicate participation was identified by matching participant identity information (name or hospital record number), the Credamo user identifier, and the device IP address across submissions; any repeated submission by the same individual was retained only once, with subsequent submissions discarded. Research fatigue was defined as a reduced capacity or willingness to engage meaningfully with the survey because of recent, repeated exposure to similar psychological questionnaire batteries. Research fatigue was assessed through (a) a brief screening item administered before the questionnaire (“Have you completed this or a very similar appearance/emotion questionnaire at our hospital within the past 12 months?”), and (b) cross-referencing the participant against the hospital's research-participation log. Participants who answered affirmatively or who were recorded as having recently completed a comparable survey were excluded to avoid habituation, careless responding, and carryover effects that could bias the self-report data.

The age range of 6–18 years was chosen for two reasons. First, this window corresponds to the mixed and permanent dentition stages, during which dentofacial disharmony becomes clinically evident and is most frequently diagnosed and treated, and during which body-image construction and appearance-based social comparison are developmentally salient. Second, the self-report instruments used in this study require sufficient reading comprehension and introspection, which are generally established by school age; children younger than 6 years were therefore excluded because they could not reliably complete the measures, and adults older than 18 years were excluded because they fall outside the pediatric target population and differ in developmental, psychosocial, and treatment characteristics.

Dentofacial deformity was diagnosed by board-certified orthodontic specialists in the Department of Pediatric Dentistry or the Department of Oral and Maxillofacial Surgery of the study hospital. The diagnosis was based on standardized clinical examination together with lateral cephalometric radiography and dental-cast analysis. Specifically, malocclusion was classified according to the Angle classification (Class I, II, or III), and the underlying skeletal pattern (skeletal Class I, II, or III) was determined from cephalometric measurements including the ANB angle, with additional assessment of maxillary and/or mandibular deficiency or excess, facial asymmetry, and soft-tissue profile. Clinically evident craniofacial syndromes (e.g., cleft lip and/or palate, hemifacial microsomia, or syndromic craniofacial dysostosis) and acquired facial asymmetries of non-dentofacial origin were distinguished from developmental dentofacial deformity by the treating specialist. A diagnosis was considered “confirmed” only when it was documented in the patient's medical record with the specialist's diagnostic note and the corresponding radiographic evidence; participants lacking such documentation were excluded (*n* = 8).

#### 2.1.3 Study sample

During the data collection period, the researchers approached a total of 523 potential participants. Among them, 12 patients had non-dentofacial facial deformities, 8 patients were younger than 6 years of age, and 8 patients had not received a confirmed diagnosis from an orthodontic specialist. During data cleaning, 13 questionnaires exhibiting highly patterned response regularities were excluded. Consequently, the final valid sample comprised 482 participants, yielding an effective response rate of 92.16%. The mean age of the participants was 10.95 ± 2.336 years, with 220 male patients (45.6%) and 262 female patients (54.4%). Detailed demographic information is presented in Table 1.

**Table 1:** Demographic characteristics of the participants.

Variables	Items	Frequency (*N*)	Proportion (%)
Gender	Male	220	45.6%
	Female	262	54.4%
Place of residence	Urban	459	95.2%
	Rural area	23	4.8%
Family structure	Both parents live together	466	96.7%
	Single parent family	12	2.5%
	Intergenerational raising	1	0.2%
	Reorganizing the family	3	0.6%
Caregiver educational background	Primary school and below	40	8.3%
	Junior high school–high school/secondary school	61	12.7%
	Bachelor degree or above	381	79.0%
Whether they have received education	Yes.	477	99.0%
	No.	5	1.0%
Personal monthly income level	≤ 2,000 RMB	5	1.0%
	2,001–5,000 RMB	39	8.1%
	5,001–8,000 RMB	64	13.3%
	8,001–12,000 RMB	121	25.1%
	≥12,001RMB	253	52.5%
Severity of deformity	Mild	310	64.3%
	Moderate	143	29.7%
	Severe	29	6.0%
Age	M ± SD	10.95 ± 2.336	

Prior to analysis, response quality was screened using two pre-specified criteria. A questionnaire was excluded as invalid if it met any of the following: (a) a completion time shorter than 6 min, which was established *a priori* through pilot testing as the minimum time required to meaningfully read and answer all items; or (b) a long-string index exceeding the threshold recommended for careless responding, defined as a run of ≥ 15 consecutive identical responses across the full item sequence. Thirteen questionnaires met at least one of these criteria and were removed, leaving 482 valid responses.

### 2.2 Measures tools

#### 2.2.1 Social appearance anxiety scale

Items measuring social appearance anxiety were drawn from the Social Appearance Anxiety Scale (SAAS) developed by Hart, Flora (42). The scale comprises 16 items encompassing social anxiety and negative body image, designed to assess individuals' emotional, cognitive, and behavioral concerns regarding their physical appearance. The scale was translated into Chinese and validated for cultural reliability and applicability by KONG and YANG (43). An example item reads: “Do you agree that you fear people may dislike you because of your appearance?” The scale has been widely administered among Chinese child and adolescent populations (44, 45). In the present study, the scale was used to measure social appearance anxiety in children with dentofacial deformities, with responses rated on a 7-point Likert scale ranging from 1 (strongly disagree) to 7 (strongly agree). The total score ranges from 16 to 112, with higher scores indicating more severe social appearance anxiety. In the present study, the Cronbach's alpha coefficient for this scale was 0.981, demonstrating excellent internal consistency.

#### 2.2.2 Self-stigma scale

Items measuring self-stigma were drawn from the Stigma and Self-Stigma Scale for Mental Health Problems developed by Docksey, Gray (46). The full scale consists of 42 items across six dimensions: Stigma to Others, Social Distance, Anticipated Stigma, Self-Stigma, Avoidant Coping, and Help-Seeking Behaviors. The present study adopted the Self-Stigma subscale, comprising six items, to assess self-stigma in children with dentofacial deformities. This selection was primarily informed by prior research indicating that dentofacial deformities may precipitate mental health problems such as anxiety and depression, and by the need to evaluate whether the deformity engenders negative self-evaluation. An example item reads: “If I had a mental health problem related to social appearance anxiety, I would feel ashamed.” The scale was translated into Chinese by Lai, Wei (47) using a back-translation procedure and was validated for reliability in a Chinese population. In the present study, this scale was used to measure self-stigma in children with dentofacial deformities, with responses rated on a 7-point Likert scale (1 = strongly disagree, 7 = strongly agree). The total score ranges from 6 to 42, with higher scores indicating more severe self-stigma. In the present study, the Cronbach's alpha coefficient for this scale was 0.970, demonstrating excellent internal consistency.

#### 2.2.3 Family support scale

The family support measure was derived from the Multidimensional Scale of Perceived Social Support (MSPSS) developed by Zimet, Dahlem (48). The full scale consists of 12 items across three dimensions: Family Support, Friend Support, and Significant Other Support. It was translated into Chinese and tested for cultural adaptability in Chinese populations by Yang, Xue (49). The present study employed the Family Support subscale, comprising four items, to assess perceived family support among children with dentofacial deformities. An example item reads: “I can obtain the emotional help and support I need from my parents.” Responses were rated on a 7-point Likert scale ranging from 1 (strongly disagree) to 7 (strongly agree). The total score ranges from 4 to 28, with higher scores indicating higher levels of family support. In the present study, the Cronbach's alpha coefficient for this scale was 0.892, demonstrating good internal consistency.

#### 2.2.4 Aggressive behavior scale

Items measuring aggressive behavior were drawn from the Aggression Scale developed by Orpinas and Frankowski (50). The scale consists of 11 items encompassing dimensions of physical aggression, verbal aggression, and anger. It was translated into Chinese and validated for cultural adaptability, reliability, and validity among Chinese adolescent populations by Lam and Li (51) and Yang, Ferraz (52). Accordingly, the present study employed this scale to assess aggressive behavior in children with dentofacial deformities, with responses rated on a 7-point Likert scale (1 = strongly disagree, 7 = strongly agree). Higher scores indicate a greater propensity for aggressive behavior. In the present study, the Cronbach's alpha coefficient for this scale was 0.951, demonstrating excellent internal consistency.

### 2.3 Data analysis

Data were analyzed using SPSS 27.0 and Mplus 8.3, with the significance level set at α = 0.05. First, SPSS 27.0 was used to conduct routine preliminary analyses, including reliability analysis, common method bias testing, descriptive statistics and correlation analysis, and between-group comparisons. Next, SPSS PROCESS Model 8 was employed to test the moderated mediation model with family support as the moderator. Finally, Mplus 8.3 was used to perform latent profile analysis (LPA) to identify latent classes of social appearance anxiety and self-stigma, and one-way analysis of variance (ANOVA) was conducted to examine differences in aggressive behavior and family support across the optimal latent profiles.

## 3 Results

### 3.1 Common method bias testing

The present study employed Harman's single-factor test to examine potential common method bias. The results revealed that four factors had eigenvalues greater than 1, with the first factor accounting for 46.475% of the total variance, which did not exceed the 50% critical threshold (53). Thus, no serious common method bias is likely present in this study.

Furthermore, anonymous data collection procedures were adopted during the survey, and the principle of data confidentiality was emphasized to further mitigate the likelihood of common method bias.

### 3.2 Descriptive statistics and correlation analysis

Descriptive statistics for the core study variables are presented in Table 2. The results indicated that the mean scores for social appearance anxiety (*M* = 2.901, SD = 1.458), self-stigma (*M* = 2.595, SD = 1.434), and aggressive behavior (*M* = 2.093, SD = 1.117) among children with dentofacial deformities all fell below the scale midpoint (*M* = 4.000). Skewness values for the core variables ranged from −1.215 to 1.288, and kurtosis values ranged from −0.872 to 1.675. According to the criteria established by Kline (54), the core study variables met the assumption of normality.

**Table 2:** Descriptive statistics of the core study variables.

Variables	*M*	SD	Skewness	Kurtosis
Social appearance anxiety	2.901	1.458	0.354	−0.872
Self–stigma	2.595	1.434	0.617	−0.521
Family support	5.341	1.404	−1.215	1.449
Aggressive behavior	2.093	1.117	1.288	1.675

Subsequently, correlation analyses were conducted among social appearance anxiety, self-stigma, family support, aggressive behavior, and the relevant demographic variables, as displayed in Table 3. The results demonstrated the following: social appearance anxiety was significantly and strongly positively correlated with self-stigma (*r* = 0.847, *p* < 0.01); social appearance anxiety was not significantly correlated with family support (*r* = 0.018, *p* > 0.05); social appearance anxiety was significantly and strongly positively correlated with aggressive behavior (*r* = 0.615, *p* < 0.01); self-stigma was not significantly correlated with family support (*r* = −0.031, *p* > 0.05); self-stigma was significantly and strongly positively correlated with aggressive behavior (*r* = 0.688, *p* < 0.01); and family support was significantly and negatively correlated with aggressive behavior (*r* = −0.120, *p* < 0.01). The variance inflation factor (VIF) values ranged from 1.008 to 3.569, all falling below the threshold of 5, indicating the absence of serious multicollinearity.

**Table 3:** Correlation matrix of the core study variables and demographic characteristics.

Variables	1	2	3	4
1. Appearance anxiety	1			
2. Self–stigma	0.847^**^	1		
3. Family support	0.018	−0.031	1	
4. Aggressive behavior	0.615^**^	0.688^**^	−0.120^**^	1

^**^p < 0.01.

### 3.3 Between-group analysis

Between-group comparisons of demographic variables with respect to aggressive behavior were subsequently conducted, as shown in Table 4. Independent-samples t-tests were used for binary variables, and one-way analysis of variance (ANOVA) was used for multi-category variables. The results indicated significant differences in aggressive behavior as a function of gender (*t* = −2.159, *p* = 0.031), educational attainment (*t* = 2.459, *p* = 0.014), place of residence (*t* = −2.102, *p* = 0.036), family structure (*F* = 6.245, *p* < 0.001), and caregiver educational background (*F* = 3.714, *p* = 0.025). However, personal monthly income level and perceived illness severity did not yield significant differences in aggressive behavior (*p* > 0.05).

**Table 4:** Between-group comparisons of demographic variables on aggressive behavior.

Variables	Items	*M*	SD	*t*/*F*	*p*	Cohen's *d*/η^2^
Gender	Male	1.974	1.103	−2.159	0.031	−0.197
	Female	2.193	1.121			
Whether they have received education	Yes.	3.309	0.980	2.459	0.014	1.106
	No.	2.080	1.112			
Place of residence	Urban	2.069	1.110	−2.102	0.036	−0.449
	Rural area	2.569	1.183			
Family structure	Both parents live together	1.614	0.632	6.245	< 0.001	0.038
	Single parent family	6.000	0.001			
	Intergenerational raising	2.089	1.108			
	Reorganizing the family	3.333	1.155			
Caregiver educational background	Primary school and below	2.011	1.061	3.714	0.025	0.015
	Junior high school–high school/secondary school	2.455	1.303			
	Bachelor degree or above	2.044	1.083			
Personal monthly income level	≤ 2,000 RMB	2.436	1.457	0.51	0.729	0.004
	2,001–5,000 RMB	2.219	1.069			
	5,001–8,000 RMB	2.003	0.935			
	8,001–12,000 RMB	2.159	1.306			
	≥12,001RMB	2.058	1.066			
Severity of deformity	Mild	2.003	1.082	2.868	0.058	0.12
	Moderate	2.260	1.152			
	Severe	2.235	1.239			

Age-stratified supplementary analysis. To explore developmental heterogeneity, participants were divided into three developmental bands: childhood (6–9 years, *n* = 141), early adolescence (10–13 years, *n* = 272), and mid-adolescence (14–18 years, *n* = 69). One-way ANOVAs revealed significant between-band differences in social appearance anxiety (*F* = 3.924, *p* = 0.020), self-stigma (*F* = 5.889, *p* = 0.003), family support (*F* = 3.680, *p* = 0.026), and aggressive behavior (*F* = 3.874, *p* = 0.021). The association between social appearance anxiety and aggressive behavior was significant and comparable in magnitude across all three bands (childhood *r* = 0.588; early adolescence *r* = 0.635; mid-adolescence *r* = 0.574; all *p* < 0.001), as was the association between self-stigma and aggressive behavior (*r* = 0.640, 0.678, and 0.798, respectively; all *p* < 0.001). By contrast, the inverse association between family support and aggressive behavior attenuated monotonically from childhood (*r* = −0.184, *p* = 0.029) through early adolescence (*r* = −0.124, *p* = 0.042) to mid-adolescence (*r* = 0.076, *p* = 0.533). These analyses are exploratory owing to unequal and, in the mid-adolescence band, modest subgroup sizes.

### 3.4 Moderated mediation of family support

To examine the moderating role of family support on the mediating effect of self-stigma, PROCESS Model 8 was employed, with self-stigma specified as the mediator, social appearance anxiety as the independent variable, family support as the moderator, and aggressive behavior as the dependent variable, as presented in Table 5. Based on the results of the between-group analyses, sex, educational attainment, place of residence, family structure, and caregiver educational background were entered as control variables.

**Table 5:** Regression path coefficients for the moderated mediation model.

Regression equation		Overall fit index			Regression coefficient			
Outcome	Predictive	*R*	*R* ^2^	*F*	*B*	*t*	LLCI	ULCI
Self-stigma	Social appearance anxiety	0.853	0.728	158.018^***^	0.836	34.503	0.788	0.883
	Family support				−0.081	−2.866	−0.136	−0.025
	Family support × social appearance anxiety				−0.048	−2.641	−0.084	−0.012
Aggressive behavior	Social appearance anxiety	0.711	0.505	53.528^***^	0.129	2.697	0.035	0.223
	Self-stigma				0.422	8.720	0.327	0.517
	Family support				−0.134	−4.488	−0.193	−0.075
	Family support × Social appearance anxiety				−0.071	−3.716	−0.109	−0.034

The results of Model 1 indicated that social appearance anxiety was significantly and positively associated with self-stigma [*B* = 0.836, *p* < 0.001, 95% CI (0.788, 0.883)]; family support was significantly and negatively associated with self-stigma [*B* = −0.081, *p* = 0.004, 95% CI (−0.136, −0.025)]; and the interaction between family support and social appearance anxiety was significantly and negatively associated with self-stigma [*B* = −0.048, *p* = 0.009, 95% CI (−0.084, −0.012)].

The results of Model 2 indicated that social appearance anxiety was significantly and positively associated with aggressive behavior [*B* = 0.129, *p* = 0.007, 95% CI (0.035, 0.223)]; the interaction between social appearance anxiety and family support was significantly and negatively associated with aggressive behavior [*B* = −0.071, *p* < 0.001, 95% CI (−0.109, −0.034)]; and self-stigma was significantly and positively associated with aggressive behavior [*B* = 0.422, *p* < 0.001, 95% CI (0.327, 0.517)].

As displayed in Table 6, Figures 2, 3, the cross-sectional direct effect of social appearance anxiety on aggressive behavior varied as a function of the moderator level. At a low level of the moderator (*M* – 1 SD), social appearance anxiety exerted a significant direct effect on aggressive behavior [Effect = 0.229, SE = 0.059, 95% CI (0.114, 0.344)]; at the mean level of the moderator, the cross-sectional direct effect was 0.129 [SE = 0.048, 95% CI (0.035, 0.223)] and remained significant; at a high level of the moderator (*M* + 1 SD), the cross-sectional direct effect decreased to 0.029 [SE = 0.051, 95% CI (−0.071, 0.128)] and was no longer significant. These findings indicate that family support negatively cross-sectional moderated the direct pathway between social appearance anxiety and aggressive behavior, with the cross-sectional direct effect attenuating and ultimately disappearing as family support increased.

**Table 6:** Cross-sectional direct and indirect effects of social appearance anxiety on aggressive behavior at different levels of family support.

Conditional direct effect				
Index	Effect	SE	LLCI	ULCI
M–SD	0.229	0.059	0.114	0.344
M	0.129	0.048	0.035	0.223
M+SD	0.029	0.051	−0.071	0.128
**Conditional indirect effects**	
Index	Effect	SE	LLCI	ULCI
M–SD	0.381	0.064	0.260	0.512
M	0.352	0.063	0.233	0.482
M+SD	0.324	0.063	0.207	0.459
**Index of moderated mediation**	
	Index	BootSE	BootLLCI	BootULCI
	−0.020	0.008	−0.038	−0.005

**Figure 2:**
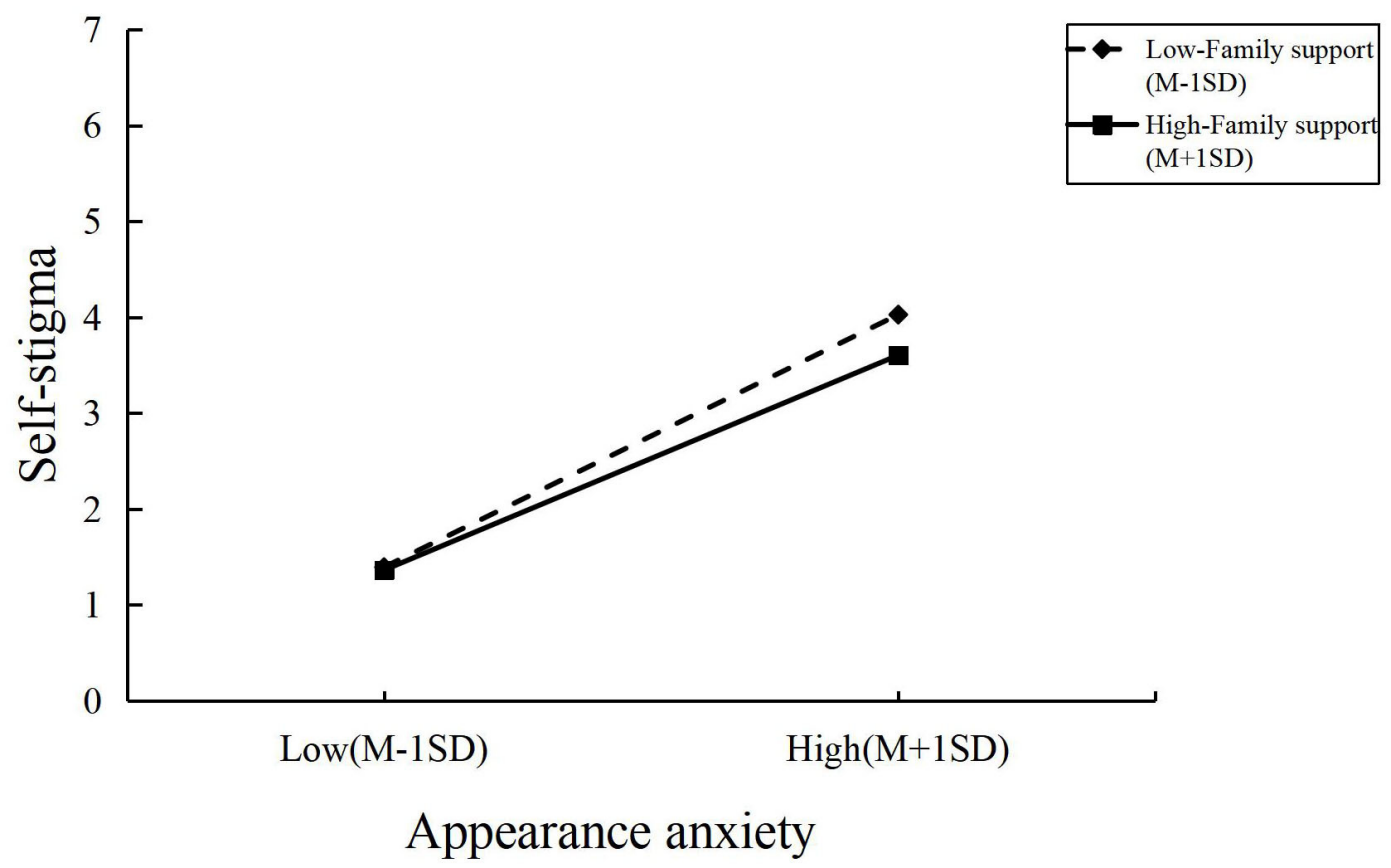
Interaction between appearance anxiety and family Support and relationship to self-stigma.

**Figure 3:**
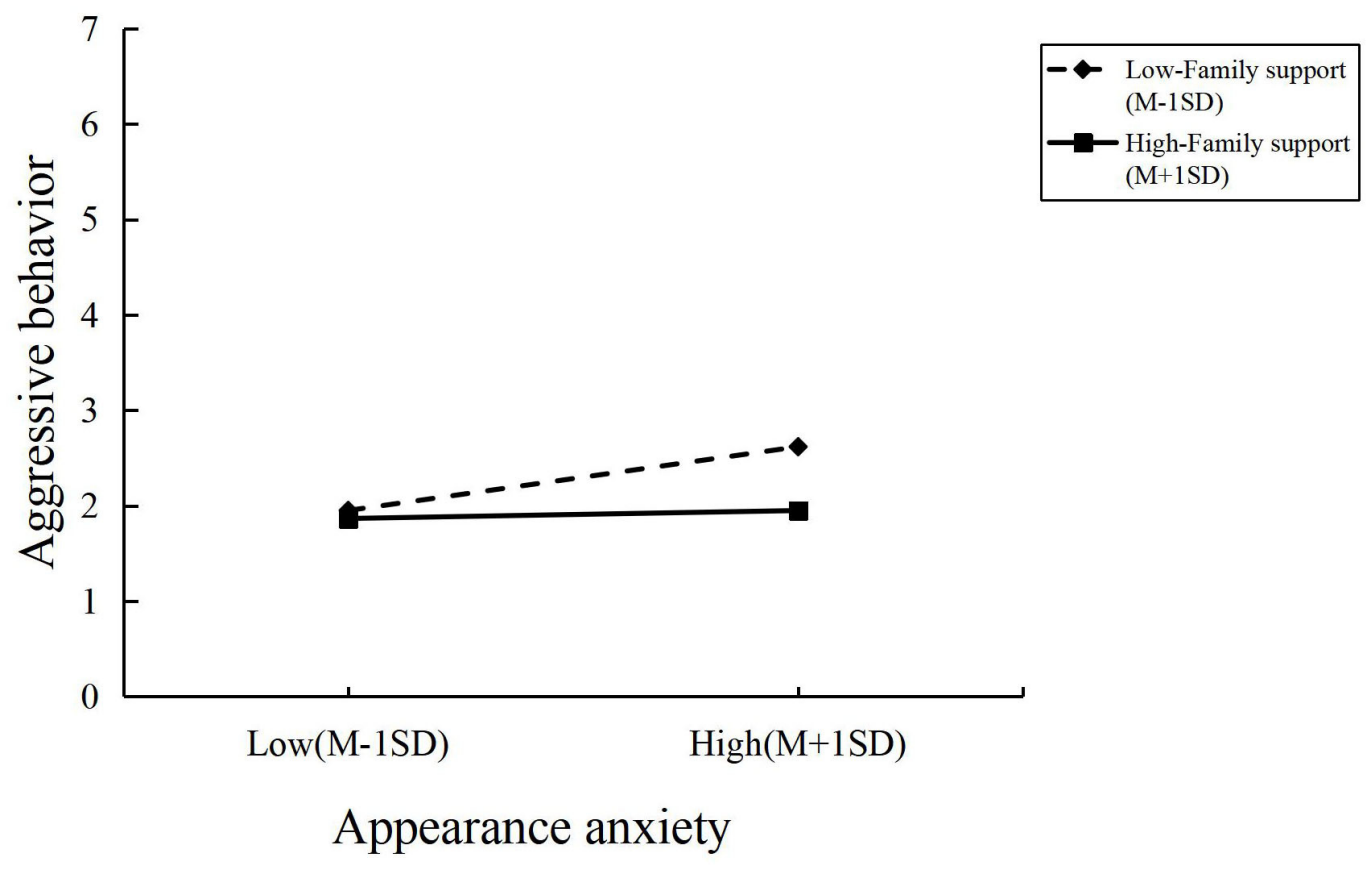
Association of appearance anxiety with family support interaction and aggressive behavior.

Across all three levels of the moderator, the indirect effect through self-stigma remained significant, although its magnitude decreased progressively as family support increased (see Table 6). Specifically, the cross-sectional indirect effect was 0.381 [Boot SE = 0.064, 95% CI (0.260, 0.512)] at low family support; 0.352 [Boot SE = 0.063, 95% CI (0.233, 0.482)] at average family support; and 0.324 [Boot SE = 0.063, 95% CI (0.207, 0.459)] at high family support. Although the cross-sectional indirect effect was significantly positive under all conditions, its magnitude decreased slightly as the level of family support increased.

The index of cross-sectional moderated mediation was −0.020 [Boot SE = 0.008, 95% bootstrap CI (−0.038, −0.005)]. This confidence interval did not contain zero, indicating a significant moderated mediation effect. The negative index suggests that family support negatively affects the strength of the mediation pathway: the higher the level of family support, the weaker the indirect effect of social appearance anxiety on aggressive behavior through self-stigma. Taken together with the monotonically decreasing trend of the conditional indirect effects, it can be concluded that family support significantly attenuated the mediating transmission effect.

### 3.5 Latent profile analysis of social appearance anxiety and self-stigma

To further delineate the latent classes of social appearance anxiety and self-stigma, Mplus 8.3 was used to construct latent profile models with one to five classes, with the items of the social appearance anxiety and self-stigma scales serving as indicators. The model fit indices are presented in Table 7.

**Table 7:** Model fit indices for latent profile models with one to five classes.

Profile	AIC	BIC	aBIC	Entropy	LMR(P)	BLRT(P)	Smallest proportion per class
1	40,395.060	40,578.889	40,439.237	–	–	–	–
2	31,793.933	32,073.855	31,861.203	0.982	< 0.001	< 0.001	56.22%/43.78%
3	29,751.565	30,127.580	29,841.928	0.994	0.007	< 0.001	51.79%/36.52%/11.68%
4	28,104.947	28,577.055	28,218.403	0.984	< 0.001	< 0.001	28.01%/30.71%/30.29%/10.99%
5	27,505.530	28,073.730	27,642.078	0.980	0.578	< 0.001	28.22%/29.67%/24.69%/7.68%/9.75%

As the number of classes increased, the information criteria (AIC, BIC, and aBIC) decreased continuously, indicating progressive improvement in model fit. Entropy values for the two- through five-class models all exceeded 0.80, suggesting acceptable classification precision across these solutions. Among them, the three-class model exhibited the highest entropy value (Entropy = 0.994), reflecting exceptionally high classification precision. Concurrently, the Lo–Mendell–Rubin test (LMRT) for the three-class model yielded a *p*-value below 0.05, indicating that the three-class solution significantly improved model fit over the two-class model. Accordingly, the three-class latent profile model was retained as the most parsimonious and well-fitting solution for the data.

To further verify the accuracy of classification regarding social appearance anxiety and self-stigma, latent class probabilities were examined, as presented in Table 8. The average posterior probabilities for each latent profile ranged from 0.997 to 0.999, all well above the recommended threshold of 0.80, indicating a high degree of classification reliability.

**Table 8:** Average posterior probability and classification probability matrix of latent profiles.

Class	Class 1	Class 2	Class 3	Average posterior probability
Class 1	0.998	0.002	0.001	0.998
Class 2	0.001	0.997	0.002	0.997
Class 3	0.001	0.001	0.999	0.999

The values in each row of the table represent the average posterior probability that an individual assigned to the profile corresponding to that row actually belongs to the underlying profile in each column. The diagonal bold values are the AvePP for each profile, reflecting the aggregated agreement within the categories.

Subsequently, Origin 2021 was used to plot the profile plot of the three-class latent profile model, as illustrated in Figure 4. Based on the profile characteristics, Class 1 was labeled the “low social appearance anxiety–self-stigma group” (51.8%); Class 2 was labeled the “moderate social appearance anxiety–self-stigma group” (36.5%); and Class 3 was labeled the “high social appearance anxiety–self-stigma group” (11.7%).

**Figure 4:**
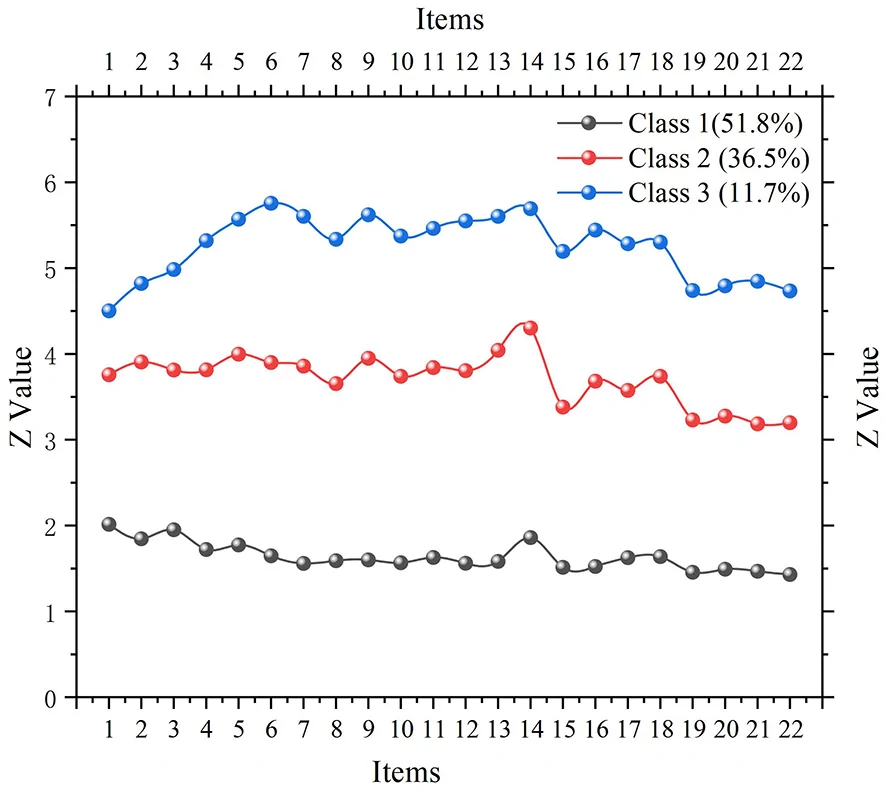
Optimal latent profile profile of appearance anxiety and self-stigma. Class 1 = low social appearance anxiety–self-stigma group (51.8%); Class 2 = moderate social appearance anxiety–self-stigma group(36.5%); Class 3 = high social appearance anxiety–self-stigma group (11.7%).

### 3.6 Differences in aggressive behavior and family support across the optimal latent profiles

To further examine the external validity of the optimal latent profiles of social appearance anxiety and self-stigma, and to compare differences in aggressive behavior and family support across the profiles, a one-way ANOVA was conducted with the three latent classes as the grouping variable and aggressive behavior and family support as dependent variables, as shown in Table 9. The results revealed significant differences across the latent profiles in both aggressive behavior (*F* = 113.374, *p* < 0.001) and family support (*F* = 3.480, *p* = 0.032), as presented in Table 9. Specifically, the high social appearance anxiety–self-stigma group exhibited the highest levels of aggressive behavior (*M* = 3.078, SD = 1.470) and family support (*M* = 5.665, SD = 0.830).

**Table 9:** ANOVA of the optimal latent profile model on aggressive behavior and family support.

Variables	Class	M	SD	F	*p*	η^2^
Aggressive behavior	Class 1	1.497 ^a^	0.618	113.374	< 0.001	0.321
	Class 2	2.626 ^a^	1.051			
	Class 3	3.078 ^a^	1.470			
Family support	Class 1	5.405 ^a^	1.669	3.480	0.032	0.014
	Class 2	5.146 ^b^	1.077			
	Class 3	5.665 ^b^	0.830			

In post hoc test analysis, two groups with the same superscript represent significant differences, and two groups with different superscripts represent no significant differences.

*Post hoc* multiple comparisons were subsequently conducted using the Bonferroni correction. The results indicated that for aggressive behavior, significant differences persisted among all three groups (*p* < 0.001). With respect to family support, no significant difference was found between Class 1 and Class 2 [*p* = 0.181, 95% CI (−0.072, 0.589)], nor between Class 1 and Class 3 [*p* = 0.625, 95% CI (−0.756, 0.236)]; however, a significant difference was observed between Class 2 and Class 3 [*p* = 0.048, 95% CI (−1.038, −0.004)].

## 4 Discussion

### 4.1 Social appearance anxiety and aggressive behavior in children with dentofacial deformities

This study empirically established the significant association between social appearance anxiety and aggressive behavior in children with dentofacial deformities. Departing from traditional theories of aggressive behavior, the present study places somatic social evaluation at the core of child aggression research (55). Social appearance anxiety is conceptualized as an internalized, persistent perception of appearance-related social threat (56), rooted in children's painful awareness that their facial features deviate from societal aesthetic norms and in their anticipated fear of negative evaluation. This anxiety continuously activates the individual's threat-monitoring system, prompting children to adopt aggressive behavior as a preemptive defense strategy or as an outlet for pent-up frustration — in essence, a maladaptive social survival strategy. This finding substantially extends the biopsychosocial model of aggression by highlighting that somatic appearance, as a biological factor, may ultimately manifest as aggressive behavior as a social consequence under the pressure of specific social evaluation contexts.

### 4.2 The cross-sectional mediating role of self-stigma

This study elucidates the cross-sectional mediating role of self-stigma in the relationship between social appearance anxiety and aggressive behavior among children with dentofacial deformities, thereby enriching the application of self-stigma theory to this special population. Specifically, external facial differences are first perceived by the individual as anxiety, constituting a primary stressor (57). This anxiety, in turn, catalyzes the active internalization of socially devaluing beliefs, giving rise to self-stigma—the conviction that one is inferior, defective, and blameworthy by reason of one's appearance. As a potent secondary stressor, self-stigma engenders profound psychological distress and social dysfunction through multiple psychological mechanisms, including diminished self-esteem, shame, and the anticipation of social rejection (36). Consequently, aggressive behavior emerges as a means of discharging accumulated shame and anger, countering a perceived hostile environment, or regaining a sense of control (58). This mediation model provides the first empirical validation, within a pediatric dentofacial deformity population, of the central pathological role of appearance-related self-stigma, thereby refining the theoretical framework of the anxiety–stigma–maladaptive behavior pathway. Crucially, it emphasizes that children do not passively endure stigma; rather, they actively participate in the process of translating social prejudice into self-devaluing cognitions, a process that, occurring during a critical developmental window, has the capacity to shape long-term psychological trajectories. This finding offers a theoretical reference for understanding the psychopathology of marginalized child populations.

### 4.3 The cross-sectional moderating role of family support

The significant cross-sectional moderating effect of family support on the mediating pathway of self-stigma represents an important contribution of this study to family systems theory and social-ecological theory in the context of dentofacial deformities. It empirically establishes family support as a critical contextual protective factor that effectively buffers the detrimental impact of self-stigma on psychosocial adaptation. High family support exerts its effects through multiple mechanisms. On the one hand, the unconditional love and acceptance offered by family members directly challenge children's internalized sense of low self-worth (59), providing a secure haven that helps children adopt a more rational perspective on their appearance, focus on their intrinsic abilities and values, and counteract distorted self-labels. On the other hand, family members guide children in coping with external ridicule, furnish emotional buffering and strategic support, and facilitate access to professional treatment and social resources (60). This finding corroborates the powerful capacity of the family to modulate the individual-level impact of the broader social system.

A counterintuitive result warrants careful interpretation: the high social appearance anxiety–self-stigma profile also reported the highest family support, whereas the moderate profile reported the lowest. Notably, this association was statistically weak, so the “paradox” rests on a small effect and should not be over-interpreted. Several non-mutually-exclusive explanations are nevertheless plausible. First, reactive (compensatory) family support: families may mobilize additional emotional and instrumental support precisely in response to a child's visible distress and stigmatizing experiences, such that the most anxious and self-stigmatized children elicit the most support—a reverse-causation pattern that cannot be resolved cross-sectionally. Second, overprotective or enmeshed parenting: heightened parental monitoring and intrusion may co-occur with high child-reported support while simultaneously reinforcing the child's sense of vulnerability and dependence, attenuating the protective function that support is assumed to serve. Third, measurement limitations: family support was assessed by child self-report, and children with high anxiety may perceive support differently; moreover, the family-support subscale was markedly negatively skewed, restricting variance and limiting the capacity to detect between-profile differences. This ceiling effect is itself a measurement limitation. Finally, the family-support differences may partly reflect the profile composition. Future studies should distinguish functional from overprotective support, obtain multi-informant (parent- and clinician-rated) measures, and test these mechanisms longitudinally

### 4.4 Heterogeneity analysis of social appearance anxiety and self-stigma

Through latent profile analysis, this study classified children with dentofacial deformities into three distinct latent subgroups with respect to social appearance anxiety and self-stigma: the high social appearance anxiety–self-stigma group, the moderate social appearance anxiety–self-stigma group, and the low social appearance anxiety–self-stigma group. This finding demonstrates that individuals differ markedly in the co-occurrence patterns of social appearance anxiety and self-stigma. Such heterogeneity may stem from individual differences in psychological adaptation strategies when confronting appearance defects, variations in the social environment, and disparities in personal intrinsic characteristics. The results of this study extend the traditional unidimensional measurement approach and underscore the necessity of accounting for inter-individual diversity when seeking to understand social appearance anxiety and self-stigma. Identifying these heterogeneous classes can provide a more precise theoretical basis for clinical intervention, thereby enabling the development of individualized intervention strategies tailored to subgroups with distinct psychological profiles.

Further analyses revealed that children in the high social appearance anxiety–self-stigma group tended to exhibit pronounced negative affect and social withdrawal behaviors, which may be associated with heightened attention to their appearance defects and excessive sensitivity to social evaluation. This co-occurrence pattern likely exacerbates children's psychological distress, thereby impairing their social functioning and mental health development. Children in the moderate social appearance anxiety–self-stigma group, by contrast, displayed a certain degree of psychological adjustment difficulty, yet their emotional states were comparatively stable, possibly owing to stronger psychological regulation capacities or more robust social support networks. In marked contrast, children in the low social appearance anxiety–self-stigma group exhibited relatively healthy psychological states, which may be attributable to stronger self-acceptance or lower social sensitivity. These findings suggest that psychological interventions should be tailored to the specific needs of different subgroups. For instance, the high social appearance anxiety–self-stigma group may benefit from more intensive emotional support and cognitive intervention, whereas the moderate group may require a focus on enhancing psychological resilience and social skills.

The heterogeneity analysis in this study also revealed the intrinsic interrelatedness of social appearance anxiety and self-stigma. Whereas prior studies have predominantly adopted unidimensional measurement approaches, tending to treat social appearance anxiety and self-stigma as variables related in a linear or unidirectionally causal manner, the present study, through latent profile analysis, uncovered their co-occurrence patterns across different subgroups. This finding suggests that social appearance anxiety and self-stigma may form a vicious cycle in certain individuals, whereby anxiety triggered by appearance defects further deepens self-stigmatizing tendencies, and vice versa. This cycle may intensify individuals' psychological distress. Particularly among children with dentofacial deformities, the salience of their facial features may render them more susceptible to becoming targets of social evaluation, thereby further reinforcing negative psychological states.

### 4.5 Clinical implications

As an emotional disorder, social appearance anxiety not only impairs children's social interactions but may also precipitate negative affect in response to external evaluation. The present findings indicate that the higher the level of social appearance anxiety, the more pronounced children's aggressive behavior is likely to be. Accordingly, when treating children with dentofacial deformities, clinicians should attend not only to the amelioration of the physical deformity but also to the patient's mental health status, with particular emphasis on the assessment of and intervention for social appearance anxiety. Clinical approaches such as psychological counseling and behavioral therapy can assist children in modifying negative cognitions regarding their own appearance, thereby reducing the incidence of aggressive behavior. Moreover, parents and educators should provide greater psychological support and positive reinforcement throughout children's development, helping them construct a positive self-image. This benefits not only their mental health but also their capacity for social adaptation.

Through the internalization of negative self-attributes, self-stigma can further exacerbate an individual's anxiety and maladaptive behavior. Clinically, when children accept the notion of their own appearance defects, they are prone to developing self-derogatory emotions, which not only intensify their social appearance anxiety but may also lead, via internalized negative self-evaluation, to a greater expression of aggressive behavior. Hence, clinical intervention should prioritize modifying children's self-evaluative patterns and assisting them in cultivating a positive self-image. Psychotherapeutic approaches, such as cognitive-behavioral therapy, can be employed to help children identify and challenge negative self-evaluations and learn more constructive forms of self-talk. Clinicians may also intervene at the family and school levels to strengthen children's social support systems and mitigate the formation of self-stigma. Such strategies can not only effectively reduce the incidence of social appearance anxiety and aggressive behavior, but also promote children's overall psychological wellbeing.

The family constitutes a critical environment for children's development, and the support and encouragement provided by family members can significantly shape children's self-evaluation and mental health. When family support is insufficient, children are more susceptible to developing self-stigma in response to their appearance defects, thereby intensifying their social appearance anxiety and aggressive behavior. In clinical intervention, practitioners should attend closely to the role of family support and design effective family-based intervention strategies in collaboration with parents. For example, parent training programs can equip parents with skills for providing positive reinforcement and praise, helping children build a positive self-image. Family therapy may also serve as an effective intervention modality, fostering more supportive and understanding parent–child relationships. Such approaches can not only attenuate the formation of self-stigma but also facilitate children's overall mental health and social adaptive development.

The latent profile analysis conducted in this study identified three co-occurring latent classes of social appearance anxiety and self-stigma among children with dentofacial deformities. This tripartite classification can assist clinicians in more precisely evaluating the psychological health status of children with dentofacial deformities, thereby enabling the formulation of more individualized psychological intervention strategies. For children in the high social appearance anxiety–self-stigma group, clinicians may prioritize intensive psychotherapy aimed at reconstructing self-image and reducing self-stigma. For those in the moderate social appearance anxiety–self-stigma group, a combination of psychological counseling and behavioral therapy may be applied to facilitate the gradual improvement of self-evaluation and behavioral expression. For children in the low social appearance anxiety–self-stigma group, psychological support and resource provision may be employed to further enhance their psychological wellbeing. Such a stratified approach enables clinicians to implement psychological interventions with greater efficiency, thereby promoting the comprehensive development of mental health and social adaptation among children with dentofacial deformities.

### 4.6 Limitations and future research directions

Although this study has elucidated the underlying mechanisms linking social appearance anxiety and aggressive behavior in children with dentofacial deformities, several significant methodological limitations remain. First, the cross-sectional design precludes the establishment of causal relationships among the variables; for example, the mediating role of self-stigma in the anxiety–aggression pathway may be subject to bidirectional influences.

Second, the issue of sample homogeneity is prominent: the data were collected exclusively from a tertiary stomatological hospital in Beijing, and the participants were predominantly from urban, middle-to-high-income households, with no inclusion of patients with comorbid psychiatric conditions. This limits the generalizability of the findings, and their applicability to rural, low-income, or more severely affected patient populations remains uncertain.

Third, the reliance on self-report scales may have introduced social desirability bias; for instance, children may have underreported aggressive behavior or overreported family support. Future research should adopt prospective cohort designs to track the dynamic evolution of anxiety and behavior over time, combined with multi-center sampling to enhance ecological validity. Concurrently, objective behavioral indicators (e.g., teacher ratings, experimental-observational paradigms) and physiological markers (e.g., cortisol levels) should be integrated to overcome the limitations inherent in self-report data.

Although the present model validated the moderating role of family support, other critical environmental and individual factors remain unexamined. The school support system and the quality of peer relationships may affect the self-stigma pathway, and the absence of these variables renders the analysis of variable relationships incomplete.

Fourth, although an exploratory age-stratified analysis was conducted, the three developmental bands were unequal in size (141 aged 6–9 years, 272 aged 10–13 years, and only 69 aged 14–18 years), limiting statistical power; the wide age range (6–18 years) nevertheless encompasses heterogeneous developmental stages that likely differ in the expression and behavioral consequences of appearance anxiety. Future studies with larger samples should conduct formal multi-group or age-stratified mediation and profile analyses, incorporate pubertal timing and social-media exposure, and consider objective behavior ratings.

The removal of 13 low-quality responses raises the possibility of a modest selection bias. Because these exclusions were driven by response-quality indicators rather than by respondent demographic or clinical characteristics, and because the number excluded was small, the risk of a material distortion of the sample composition is low. Consistent with this, careless or straight-line responding tends to attenuate rather than inflate true associations, so its removal is expected to reduce measurement error. Nevertheless, we cannot fully exclude the possibility that inattentive responding covaried with unmeasured respondent characteristics, and the exclusion of these cases may marginally limit generalizability. This is acknowledged as a limitation, and future studies are encouraged to incorporate attention-check items and adaptive administration to further safeguard data quality.

A further limitation is the absence of detailed clinical characterization. Dentofacial deformity is a heterogeneous category encompassing clinically distinct conditions—including cleft lip and/or palate, skeletal Class II and Class III malocclusion, and craniofacial syndromes—and psychological outcomes are known to differ across these diagnoses. The present study recorded only the confirmation of a dentofacial-deformity diagnosis by an orthodontic specialist and the participant's self-rated severity; specific diagnostic subtypes, Angle/skeletal classification, and objective severity indices were not collected. Consequently, we were unable to examine whether the observed associations vary by diagnosis, and the findings should not be interpreted as applying uniformly across all dentofacial phenotypes. Future studies should collect detailed clinical data to enable diagnosis-specific subgroup analyses

## 5 Conclusion

This study provides novel empirical evidence that social appearance anxiety was significantly associated with aggressive behavior in children with dentofacial deformities, with self-stigma showing a statistically significant indirect association and family support acting as a statistically significant. Specifically, children who internalize negative societal evaluations of their facial appearance are more likely to develop self-stigmatizing cognitions, which in turn heighten their propensity for aggressive behavior. High levels of family support attenuate both the direct anxiety–aggression pathway and the indirect pathway transmitted through self-stigma, underscoring the family's pivotal role as a contextual buffer in this vulnerable population. Furthermore, the identification of three distinct latent profiles of social appearance anxiety and self-stigma reveals substantial inter-individual heterogeneity, with the high-profile group exhibiting the most elevated levels of aggressive behavior. The results highlight the necessity of embedding routine psychological screening for social appearance anxiety and self-stigma within pediatric dental and orthodontic care, and of developing stratified, family-involved interventions tailored to distinct psychological profiles.

## Data Availability

The raw data supporting the conclusions of this article will be made available by the authors, without undue reservation.
